# Editorial Note: Honokiol Inhibits Non-Small Cell Lung Cancer Cell Migration by Targeting PGE_2_-Mediated Activation of β-Catenin Signaling

**DOI:** 10.1371/journal.pone.0303600

**Published:** 2024-05-13

**Authors:** The PLOS ONE Editors

Following the publication of this article [1], concerns were raised regarding results presented in Fig 2. Specifically, the Fig 2C H1299 Honokiol 0μM and the Fig 2C H1299 Honokiol 10μM panels appear similar.

The corresponding author commented that they were unable to retrieve the original data underlying the Fig 2 results. In the absence of these data, the concerns with Fig 2 cannot be fully resolved, and the Fig 2 results should be interpreted with caution.

The *PLOS ONE* Editors issue this Editorial Note to notify readers of the unresolved issue with Fig 2 and to relay to readers that the original data underlying this study may no longer be available.
